# Trends in Mental, Behavioral, and Developmental Disorders Among Children and Adolescents in the US, 2016–2021

**DOI:** 10.5888/pcd21.240142

**Published:** 2024-12-12

**Authors:** Rebecca T. Leeb, Melissa L. Danielson, Angelika H. Claussen, Lara R. Robinson, Lydie A. Lebrun-Harris, Reem Ghandour, Rebecca H. Bitsko, Samuel M. Katz, Jennifer W. Kaminski, Jessica Brown

**Affiliations:** 1 National Center on Birth Defects and Developmental Disabilities, Centers for Disease Control and Prevention, Atlanta, Georgia; 2 Maternal and Child Health Bureau, Health Resources and Services Administration, Rockville, Maryland; 3 Oak Ridge Institute for Science and Education, Oak Ridge, Tennessee; 4 Office of Policy, Performance, and Evaluation, Centers for Disease Control and Prevention, Atlanta, Georgia; 5 National Center for HIV/AIDS, Viral Hepatitis, STD, and TB Prevention, Centers for Disease Control and Prevention, Atlanta, Georgia

## Abstract

**Introduction:**

Childhood mental, behavioral, and developmental disorders (MBDD) are common and are associated with poor health and well-being. Monitoring the prevalence of MBDDs among children and factors that may influence health outcomes is important to understanding risk and promoting population health.

**Method:**

We examined trends in parent-reported lifetime MBDDs among children and associated health promotion and risk indicators from 2016 through 2021 by using data from the National Survey of Children’s Health. Estimates of prevalence and average annual percentage change were stratified by specific MBDDs and demographic characteristics (eg, sex, age, race and ethnicity). Children with any MBDDs versus none were compared overall and by MBDD subgroup on health care, family, and community indicators.

**Results:**

From 2016 through 2021, MBDD prevalence among children aged 3 to 17 years increased from 25.3% to 27.7%; increases were specific to anxiety, depression, learning disability, developmental delay, and speech or language disorder. Unmet health care needs increased annually by an average of approximately 5% among children with MBDDs. Each year from 2016 to 2021, approximately 60% of children with MBDDs received mental or developmental services in the past 12 months. Each year, a higher percentage of parents of children with MBDDs compared with children without MBDDs reported poor mental health (14.7% MBDD, 5.7% no MBDD) and economic stress (21.6% MBDD, 11.5% no MBDD).

**Conclusion:**

Increasing prevalence of certain MBDDs and MBDD-associated indicators, before and during the COVID-19 pandemic, highlights the need for improved pediatric mental health training for health care providers, for prevention and intervention efforts, and for policies addressing economic stability and equitable access to mental health services.

**Summary**

**What is already known on this topic?**

Childhood mental, behavioral, and developmental disorders (MBDD) are common and are associated with poor health and well-being.

**What is added by this report?**

We examined trends in parent-reported lifetime MBDDs among children and associated health promotion and risk indicators from 2016 through 2021 by using a nationally representative sample to demonstrate increases in specific MBDDs and changes in risk and protective indicators.

**What are the implications for public health practice?**

Findings suggest opportunities to improve pediatric mental health training for health care providers, for prevention and intervention efforts, and for policies addressing economic stability and equitable access to mental health services.

## Introduction

Childhood mental, behavioral, and developmental disorders (MBDD) are common and are associated with immediate and long-term risk for poor physical health, chronic disease, and decreased educational attainment and economic outcomes (1). MBDDs are also associated with sociodemographic and contextual characteristics (eg, community safety and support) and other social determinants of health that influence access to health care, safe environments, and quality of life outcomes (1–3).

Monitoring the prevalence of MBDDs among children and the associated factors that may influence health outcomes can help draw attention to early risk and health promotion factors that may affect population health. MBDDs include mental, emotional and behavioral disorders (ie, attention deficit/hyperactivity disorder [ADHD], behavioral/conduct problems, anxiety problems, depression, Tourette syndrome) and developmental, learning, and language disorders (ie, autism spectrum disorder [ASD], learning disorders, intellectual disability, developmental delay, and speech and other language disorders) (2,4,5). MBDDs often co-occur and share risk and protective factors (2,6).

The COVID-19 pandemic influenced social determinants of health and exacerbated many stressors associated with MBDDs in children. Research findings suggest that risk factors for MBDDs increased during the pandemic, including evidence of worsening adult mental health, decreases in parents’ reported ability to cope with the demands of raising children, increases in household financial stress, unmet needs for child health care, and barriers to early identification of developmental delays and disabilities in young children (7–9). The COVID-19 pandemic also introduced expanded opportunities for health care and mental health care access through telehealth, support to families and communities to offset health and economic effects of the pandemic, and changes that could promote resilience (eg, technological opportunities to increase social connection, new workplace flexibilities), potentially mitigating some of the pandemic-related risks to children’s mental health and well-being (8).

Information is limited about trends in the prevalence of MBDDs and related health promotion and risk indicators over the period before and during the pandemic. We describe trends in childhood MBDDs and associated health care, family, and community indicators in a nationally representative sample, from 2016 through 2021, to identify potential factors to consider for focused efforts by health care and public health professionals to support children’s and parents’ mental health and to improve access to resources and interventions.

## Methods

We examined trends in MBDDs among children and associated health promotion and risk indicators from 2016 through 2021 by using data from the National Survey of Children’s Health (NSCH). NSCH is an annual, nationally representative, cross-sectional survey of parents and guardians, funded and directed by the Health Resources and Services Administration’s Maternal and Child Health Bureau and conducted by the US Census Bureau. Parents of children aged 0 to 17 years living in noninstitutional settings in all 50 states and the District of Columbia responded to an address-based survey by mail or web (10).

NSCH’s annual weighted response rates for 2016 through 2021 ranged from 37.4% to 43.1%, and the annual interview completion rates among screened households known to contain a child ranged from 69.7% to 81.2% (11). The analytic sample was restricted to children aged 3 to 17 years for this study. Children were excluded from analyses if they had missing data on more than 3 MBDD items (0.07% of our study sample). The final analytic sample for our study was 193,877 children aged 3 to 17 years.

### Mental, behavioral, and developmental disorders

Parents were asked whether a doctor or other health care provider ever told them that their child had any of a series of disorders, including attention deficit/hyperactivity disorder (ADHD), behavioral and conduct problems, anxiety problems, depression, Tourette syndrome, autism spectrum disorder (ASD), learning disability, intellectual disability, developmental delay, or speech or other language disorders. For some disorders (behavioral or conduct problems, developmental delay, intellectual disability, learning disability, speech or other language disorder) the stem question included “educator”: “Has a doctor, other health care provider, or educator ever told you that this child has (specified disorder)?” (Appendix).

We grouped disorders into 2 subgroups: 1) mental, emotional, and behavioral disorders (MEB) (ADHD, behavioral and conduct problems, anxiety problems, depression, and Tourette syndrome) and 2) developmental, learning, and language disorders (DLLD) (ASD, learning disability, intellectual disability, developmental delay, and speech or other language disorders). MEB and DLLD categories are not mutually exclusive; therefore, children could be included in both categories.

### Demographic, health care, family, and community characteristics

We included the following parent-reported demographic characteristics of the child and family: child sex, age, race or ethnicity, household income, highest level of household education, primary household language, and urban/suburban or rural residence. Federal poverty level was calculated by the US Census Bureau based on household size and income. Multiple imputed data were incorporated into the analysis for respondents with missing data for federal poverty level (18.3% of the analytic sample). The US Census Bureau reviewed the urban/suburban or rural residence classification product for unauthorized disclosure of confidential information and approved the disclosure avoidance practices applied to this release: CBDRB-FY23-POP001–0105.

Parents also reported on health care, family, and community characteristics. Health care characteristics included in our analysis were receipt of preventive health care for their child in the past 12 months, their child’s unmet health care needs in the past 12 months, adequacy of health insurance, and their child’s receipt of mental and/or developmental services in the past 12 months. Family characteristics included in our analysis were parental mental health and household economic stress. Community characteristics included were parents’ perception of neighborhood support and neighborhood safety (Appendix). 

### Analyses

We calculated weighted prevalence estimates overall and stratified by specific MBDD diagnosis, MBDD subgroup (ie, MEB, DLLD), sex, age, race or ethnicity, household income relative to federal poverty level, highest education level in the household, primary household language, and urban/suburban or rural residence. Children with any versus no parent-reported lifetime MBDDs were compared overall and by MBDD subgroup on health care, family, and community indicators.

We conducted all analyses in SAS-callable SUDAAN, version 11.1 (RTI International) to account for the complex sample design and survey weights. We used Joinpoint (National Cancer Institute) regression with permutation tests to estimate the average annual percentage change (AAPC) and 95% CIs and linear trends in the prevalence of MBDDs and health care, family, and community indicators from 2016 through 2021. *P* < .05 was used to define significance. Point estimates across MBDD subgroups that had nonoverlapping CIs were considered significantly different.

## Results

### Prevalence of mental, behavioral and developmental disorders

From 2016 through 2021, the lifetime prevalence of MBDDs among children aged 3 to 17 years increased from 25.3% to 27.7% (AAPC = 1.8; 95% CI, 1.1–2.5) (Table 1), with significant increases among some demographic subgroups, including females (AAPC = 3.3; 95% CI, 0.2–6.5), adolescents aged 12 to 17 years (AAPC = 2.7; 95% CI, 1.0–4.5), non-Hispanic White children (AAPC = 2.3; 95% CI, 0.7–3.9), children living in households with annual incomes of 100% or less of the federal poverty level, households in which one or more caregivers had more than a high school education (AAPC = 2.9; 95% CI, 1.5–4.3), households where English was the primary language (AAPC = 2.0; 95% CI, 1.3–2.6), and children living in urban/suburban areas (AAPC = 1.9; 95% CI,1.1–2.7) (Table 2).

**Table 1:** Prevalence of Children Aged 3–17 Years With Any Lifetime Mental, Behavioral, or Developmental Disorder Diagnosis, National Survey of Children’s Health, 2016–2021

Variable	2016–2021, % (95% CI)	2016, % (95% CI)	2017, % (95% CI)	2018, % (95% CI)	2019, % (95% CI)	2020, % (95% CI)	2021, % (95% CI)	Average Annual % Change, (95% CI)	*P* value^a^
**Any MBDD (ever)** b	26.3 (25.9 to 26.8)	25.3 (24.4 to 26.2)	25.5 (24.2 to 26.9)	26.4 (25.3 to 27.5)	26.1 (25.0 to 27.2)	27.0 (26.0 to 28.0)	27.7 (26.8 to 28.7)	1.8 (1.1 to 2.5)	.002
**Any MEB**	19.5 (19.1 to 19.9)	18.6 (17.8 to 19.4)	18.3 (17.2 to 19.4)	19.2 (18.3 to 20.2)	19.7 (18.8 to 20.8)	20.4 (19.5 to 21.3)	20.6 (19.8 to 21.5)	2.3 (1.5 to 3.2)	.001
ADHD	10.0 (9.7 to 10.3)	9.9 (9.4 to 10.5)	9.7 (8.8 to 10.5)	9.9 (9.2 to 10.6)	9.6 (9.0 to 10.3)	10.2 (9.6 to 10.8)	10.6 (10.0 to 11.2)	1.3 (−0.6 to 3.4)	.13
Behavioral/conduct problems	9.0 (8.8 to 9.3)	9.4 (8.8 to 10.0)	8.9 (8.1 to 9.8)	8.7 (8.1 to 9.4)	8.6 (7.9 to 9.3)	9.8 (9.1 to 10.5)	8.9 (8.3 to 9.5)	−0.3 (−3.7 to 3.1)	.79
Anxiety problems	9.9 (9.6 to 10.2)	8.4 (7.8 to 8.9)	8.5 (7.7 to 9.2)	9.7 (9.1 to 10.4)	11.0 (10.2 to 11.8)	10.7 (10.0 to 11.3)	11.1 (10.4 to 11.7)	6.0 (2.8 to 9.2)	.006
Depression	4.7 (4.5 to 4.9)	4.2 (3.8 to 4.6)	3.8 (3.3 to 4.3)	4.8 (4.3 to 5.4)	5.0 (4.4 to 5.6)	4.9 (4.5 to 5.4)	5.4 (5.0 to 5.9)	5.3 (1.9 to 8.9)	.01
Tourette syndrome	0.3 (0.2 to 0.3)	0.3 (0.2 to 0.4)	0.2 (0.1 to 0.3)	0.2 (0.1 to 0.3)	0.4 (0.2 to 0.7)	0.2 (0.2 to 0.4)	0.3 (0.2 to 0.5)	5.3 (−6.7 to 18.9)	.30
**Any DLLD**	14.8 (14.5 to 15.2)	14.1 (13.3 to 14.8)	14.8 (13.7 to 16.0)	14.6 (13.8 to 15.5)	14.7 (13.8 to 15.6)	15.3 (14.5 to 16.1)	15.5 (14.8 to 16.3)	1.8 (1.0 to 2.6)	.004
Autism spectrum disorder	3.1 (2.9 to 3.3)	2.8 (2.5 to 3.1)	3.3 (2.6 to 4.0)	2.9 (2.5 to 3.4)	3.3 (2.8 to 3.8)	3.0 (2.6 to 3.3)	3.4 (3.0 to 3.9)	3.1 (−1.2 to 7.7)	.12
Learning disability	7.8 (7.5 to 8.1)	7.3 (6.7 to 7.8)	7.8 (6.9 to 8.7)	7.6 (7.0 to 8.3)	7.3 (6.7 to 8.0)	8.2 (7.6 to 8.9)	8.5 (7.9 to 9.2)	2.9 (0.1 to 5.9)	.05
Intellectual disability	1.2 (1.1 to 1.4)	1.1 (0.9 to 1.3)	1.4 (1.0 to 1.9)	1.2 (0.9 to 1.4)	1.0 (0.8 to 1.3)	1.5 (1.2 to 1.9)	1.3 (1.0 to 1.6)	2.8 (−5.5 to 11.8)	.41
Developmental delay	7.2 (6.9 to 7.4)	6.5 (6.0 to 7.0)	7.2 (6.5 to 8.1)	7.0 (6.4 to 7.6)	7.2 (6.6 to 7.9)	7.6 (7.0 to 8.2)	7.4 (6.9 to 8.0)	2.5 (0.4 to 4.7)	.03
Speech or other language disorder	8.8 (8.5 to 9.1)	8.2 (7.6 to 8.8)	8.5 (7.6 to 9.5)	8.7 (8.0 to 9.5)	8.8 (8.1 to 9.5)	8.9 (8.3 to 9.6)	9.7 (9.1 to 10.4)	3.0 (1.5 to 4.7)	.006

Abbreviations: ADHD, attention deficit/hyperactivity disorder; DLLD, developmental, learning, and language disorders; MBDD, mental, behavioral, and developmental disorder; MEB, mental, emotional, and behavioral condition.

a Calculated by linear trend test. Significant at *P* < .05.

b MBDD categories are not mutually exclusive. Children with multiple conditions are included in each applicable category.

**Table 2:** Demographic Characteristics and Prevalence of Any Mental, Emotional, and Behavioral (MEB) and Developmental, Learning, and Language Disorder (DLLD) Lifetime Diagnosis by Demographic Characteristics Among Children Aged 3–17 Years, National Survey of Children’s Health, 2016–2021

Variable	2016–2021, % (95% CI)	2016, % (95% CI)	2017, % (95% CI)	2018, % (95% CI)	2019, % (95% CI)	2020, % (95% CI)	2021, % (95% CI)	Average Annual Change, % (95% CI)	*P* value^a^
**Sex**									
MBDD^b^									
Male	30.8 (30.1 to 31.4)	30.0 (28.6 to 31.3)	31.3 (29.3 to 33.3)	29.9 (28.4 to 31.5)	30.9 (29.2 to 32.6)	31.5 (30.1 to 33.0)	31.0 (29.7 to 32.4)	0.7 (−0.5 to 1.9)	.17
Female	21.7 (21.2 to 22.3)	20.5 (19.3 to 21.8)	19.5 (17.9 to 21.2)	22.7 (21.2 to 24.2)	21.1 (19.8 to 22.6)	22.3 (21.0 to 23.7)	24.3 (22.9 to 25.7)	3.3 (0.2 to 6.5)	.04
MEB^c^									
Male	22.4 (21.8 to 23.0)	22.1 (21.0 to 23.4)	22.0 (20.3 to 23.8)	21.2 (19.9 to 22.5)	23.6 (22.0 to 25.2)	23.1 (21.8 to 24.4)	23.3 (21.1 to 23.4)	0.7 (−1.5 to 3.0)	.42
Female	16.4 (15.9 to 17.0)	15.0 (14.0 to 16.0)	14.3 (13.0 to 15.8)	17.1 (15.8 to 18.5)	15.7 (14.5 to 16.9)	17.6 (16.3 to 18.9)	18.9 (17.7 to 20.2)	4.8 (1.3 to 8.4)	.02
DLLD^d^									
Male	19.0 (18.5 to 19.6)	17.8 (16.7 to 19.0)	19.4 (17.7 to 21.2)	18.5 (17.2 to 19.8)	19.0 (17.6 to 20.5)	19.6 (18.4 to 20.8)	19.9 (18.7 to 21.2)	2.0 (0.5 to 3.5)	.02
Female	10.5 (10.0 to 10.9)	10.2 (9.3 to 11.2)	10.1 (8.8 to 11.5)	10.5 (9.4 to 11.7)	10.2 (9.2 to 11.3)	10.8 (9.8 to 11.8)	11.0 (10.1 to 11.9)	1.6 (0.2 to 3.0)	.03
**Age group, y**									
MBDD									
3–5	15.5 (14.7 to 16.3)	14.9 (13.1 to 16.8)	16.7 (14.2 to 19.5)	14.7 (12.8 to 16.7)	14.3 (12.4 to 16.3)	15.8 (13.9 to 17.8)	16.5 (14.9 to 18.3)	1.4 (−2.5 to 5.4)	.38
6–11	25.8 (25.1 to 26.5)	25.0 (23.5 to 26.5)	24.8 (22.6 to 27.0)	27.9 (26.1 to 29.7)	25.7 (23.9 to 27.5)	26.2 (24.6 to 27.8)	25.5 (24.0 to 27.0)	0.4 (−2.6 to 3.4)	.76
12–17	32.1 (31.4 to 32.8)	30.8 (29.4 to 32.2)	30.5 (28.5 to 32.7)	30.5 (28.7 to 32.2)	32.2 (30.3 to 34.1)	33.2 (31.6 to 34.8)	35.2 (33.6 to 36.9)	2.7 (1.0 to 4.5)	.01
MEB									
3–5	6.6 (6.1 to 7.1)	6.8 (5.4 to 8.3)	6.1 (4.8 to 7.7)	6.6 (5.5 to 8.0)	5.8 (4.6 to 7.3)	7.6 (6.2 to 9.3)	6.5 (5.5 to 7.6)	0.5 (−5.4 to 6.8)	.83
6–11	18.6 (18.0 to 19.2)	18.0 (16.7 to 19.4)	17.8 (15.9 to 19.7)	19.4 (17.9 to 21.0)	18.8 (17.3 to 20.4)	19.2 (17.8 to 20.7)	18.3 (17.0 to 19.6)	0.7 (−1.6 to 3.0)	.44
12–17	26.5 (25.9 to 27.2)	25.0 (23.7 to 26.3)	24.6 (22.8 to 26.6)	25.0 (23.5 to 26.7)	27.3 (25.5 to 29.1)	27.6 (26.1 to 29.1)	29.6 (28.1 to 31.2)	3.6 (1.6 to 5.6)	.007
DLLD									
3–5	12.8 (12.0 to 13.6)	12.0 (10.3 to 13.8)	14.2 (11.8 to 16.8)	11.7 (9.9 to 13.6)	12.6 (10.8 to 14.6)	12.6 (11.0 to 14.4)	13.7 (12.1 to 15.4)	1.6 (−3.0 to 6.4)	.40
6–11	15.7 (15.1 to 16.3)	14.5 (13.3 to 15.7)	15.9 (14.0 to 18.0)	17.0 (15.5 to 18.7)	15.4 (14.0 to 16.8)	15.6 (14.4 to 17.0)	15.9 (14.6 to 17.2)	1.1 (−2.2 to 4.5)	.41
12–17	15.0 (14.4 to 15.5)	14.7 (13.6 to 15.8)	14.0 (12.5 to 15.6)	13.6 (12.5 to 14.8)	15.1 (13.7 to 16.6)	16.2 (14.9 to 17.5)	16.1 (14.9 to 17.4)	2.8 (−0.6 to 6.3)	.09
**Race or ethnicity**									
MBDD									
American Indian or Alaska Native, non-Hispanic	31.7 (27.4 to 36.3)	30.9 (22.6 to 40.2)	21.0 (10.4 to 35.4)	31.7 (21.8 to 43.1)	34.4 (23.1 to 47.2)	30.8 (21.2 to 41.8)	40.2 (30.1 to 50.9)	5.6 (−2.9 to 14.8)	.14
Asian, non-Hispanic	11.1 (10.0 to 12.3)	10.4 (8.2 to 12.9)	12.5 (8.8 to 17.1)	12.0 (9.3 to 15.2)	11.0 (8.2 to 14.5)	10.8 (8.5 to 13.5)	10.0 (8.1 to 12.2)	−1.8 (−6.6 to 3.3)	.38
Black, non-Hispanic	26.6 (25.3 to 28.0)	28.4 (25.5 to 31.4)	27.9 (23.7 to 32.4)	24.9 (21.7 to 28.3)	25.0 (21.9 to 28.3)	28.3 (25.5 to 31.2)	25.4 (22.8 to 28.2)	−1.3 (−5.3 to 3.0)	.45
Hispanic	23.9 (22.7 to 25.1)	22.3 (19.9 to 24.9)	24.1 (20.5 to 28.0)	24.1 (21.2 to 27.2)	22.1 (19.2 to 25.2)	24.4 (21.8 to 27.2)	26.1 (23.5 to 28.8)	2.5 (−0.5 to 5.7)	.08
Native Hawaiian/Other Pacific Islander, non-Hispanic	18.2 (12.1 to 25.7)	–e	–e	–e	–e	–e	–e	−15.4 (−33.0 to 6.7)	.12
White, non-Hispanic	28.7 (28.2 to 29.1)	27.0 (26.1 to 28.0)	26.7 (25.4 to 28.0)	29.2 (28.1 to 30.4)	29.8 (28.6 to 31.0)	29.3 (28.2 to 30.3)	30.2 (29.2 to 31.3)	2.3 (0.7 to 3.9)	.02
Two or more races, non-Hispanic	29.0 (27.3 to 30.7)	28.4 (24.6 to 32.4)	28.7 (23.7 to 34.1)	27.7 (23.8 to 31.8)	26.9 (23.1 to 31.0)	29.5 (25.4 to 33.7)	32.6 (29.0 to 36.3)	2.6 (−1.2 to 6.6)	.13
MEB									
American Indian or Alaska Native, non-Hispanic	23.1 (19.4 to 27.1)	24.0 (16.5 to 32.9)	15.4 (7.8 to 26.3)	26.2 (17.0 to 37.3)	22.1 (12.7 to 34.3)	22.0 (13.5 to 32.6)	27.4 (19.0 to 37.1)	2.9 (−7.1 to 14.0)	.48
Asian, non-Hispanic	7.1 (6.2 to 8.0)	7.2 (5.3 to 9.4)	5.7 (3.5 to 8.9)	8.8 (6.4 to 11.8)	7.0 (4.7 to 9.9)	6.6 (5.1 to 8.5)	7.1 (5.4 to 9.0)	−0.8 (−8.9 to 8.0)	.81
Black, non-Hispanic	19.9 (18.7 to 21.1)	21.7 (19.0 to 24.6)	21.6 (18.0 to 25.6)	17.8 (15.2 to 20.7)	18.9 (16.1 to 21.9)	20.0 (17.6 to 22.6)	19.4 (17.0 to 22.0)	−1.9 (−6.1 to 2.4)	.28
Hispanic	14.1 (13.1 to 15.1)	13.3 (11.3 to 15.6)	15.2 (12.1 to 18.7)	14.1 (11.8 to 16.6)	12.5 (10.3 to 15.0)	13.8 (11.7 to 16.0)	15.7 (13.6 to 18.1)	2.0 (−3.3 to 7.5)	.36
Native Hawaiian/Other Pacific Islander, non-Hispanic	11.1 (6.5 to 17.5)	–e	–e	–e	–e	–e	–e	0.6 (−10.4 to 12.8)	.90
White, non-Hispanic	21.6 (21.2 to 22.0)	20.3 (19.5 to 21.2)	19.6 (18.4 to 20.8)	21.9 (20.9 to 23.0)	22.5 (21.4 to 23.6)	22.3 (21.3 to 23.3)	23.2 (22.2 to 24.1)	2.9 (1.1 to 4.7)	.01
Two or more races, non-Hispanic	21.8 (20.3 to 23.4)	21.9 (18.3 to 25.7)	22.1 (17.3 to 27.5)	20.1 (16.7 to 23.8)	19.5 (16.3 to 23.0)	22.4 (18.7 to 26.6)	24.8 (21.5 to 28.3)	2.5 (−3.1 to 8.4)	.30
DLLD									
American Indian or Alaska Native, non-Hispanic	18.7 (15.4 to 22.4)	16.2 (10.6 to 23.1)	—e	15.5 (9.3 to 23.8)	21.8 (12.9 to 33.3)	17.6 (10.9 to 26.2)	26.1 (17.5 to 36.3)	9.5 (−0.6 to 20.6)	.06
Asian, non-Hispanic	7.4 (6.4 to 8.4)	6.5 (4.8 to 8.5)	9.4 (6.1 to 13.5)	7.4 (5.5 to 9.7)	7.9 (5.4 to 11.0)	7.2 (5.3 to 9.5)	5.9 (4.5 to 7.6)	−2.7 (−11.7 to 7.3)	.48
Black, non-Hispanic	16.2 (15.1 to 17.3)	16.9 (14.6 to 19.4)	15.4 (12.2 to 19.0)	15.6 (12.9 to 18.6)	16.1 (13.5 to 18.9)	18.6 (16.2 to 21.3)	14.7 (12.7 to 16.8)	−0.7 (−6.7 to 5.6)	.75
Hispanic	14.1 (13.1 to 15.1)	13.4 (11.3 to 15.6)	15.2 (12.1 to 18.7)	14.1 (11.8 to 16.6)	12.5 (10.3 to 15.0)	13.8 (11.7 to 16.0)	15.7 (13.6 to 18.0)	1.9 (−3.4 to 7.3)	.39
Native Hawaiian/Other Pacific Islander, non-Hispanic	14.0 (8.3 to 21.7)	–e	–e	–e	–e	–e	–e	−21.7 (−44.7 to 11.0)	.12
White, non-Hispanic	15.5 (15.1 to 15.8)	14.5 (13.8 to 15.2)	14.9 (13.9 to 16.0)	15.2 (14.3 to 16.1)	16.1 (15.1 to 17.2)	15.8 (14.9 to 16.6)	16.3 (15.5 to 17.1)	2.3 (1.3 to 3.3)	.003
Two or more races, non-Hispanic	15.7 (14.5 to 17.1)	13.0 (10.8 to 15.5)	17.3 (12.7 to 22.8)	15.5 (12.6 to 18.8)	14.5 (11.7 to 17.6)	16.0 (13.3 to 19.0)	17.9 (15.2 to 21.0)	4.9 (−0.5 to 1.7)	.07
**Household income relative to federal poverty level** f, %									
MBDD									
<100	29.1 (27.8 to 30.4)	29.5 (26.9 to 32.4)	28.4 (24.6 to 32.4)	28.2 (25.2 to 31.3)	30.0 (27.0 to 33.3)	30.5 (27.4 to 33.8)	28.0 (25.0 to 31.3)	0.0 (−2.5 to 2.6)	.99
100–199	27.3 (26.2 to 28.4)	25.8 (23.4 to 28.3)	27.1 (23.9 to 30.7)	27.4 (24.7 to 30.3)	26.6 (24.0 to 29.4)	27.6 (25.1 to 30.2)	29.4 (27.0 to 32.0)	2.1 (0.4 to 3.9)	.03
200–399	26.1 (25.3 to 26.9)	24.1 (22.5 to 25.7)	24.9 (22.7 to 27.3)	26.0 (23.9 to 28.2)	25.9 (23.9 to 28.0)	27.5 (25.4 to 29.4)	27.9 (26.0 to 29.9)	3.0 (2.1 to 3.8)	.001
≥400	24.1 (23.5 to 24.8)	23.1 (21.9 to 24.3)	23.0 (21.3 to 24.7)	24.8 (23.2 to 26.6)	23.5 (22.0 to 25.1)	24.2 (22.6 to 25.8)	26.2 (24.8 to 27.6)	2.2 (0.2 to 4.3)	.04
MEB									
<100	21.2 (20.0 to 22.4)	21.5 (19.2 to 24.0)	19.7 (16.6 to 23.1)	20.8 (18.2 to 23.7)	22.2 (19.5 to 25.2)	22.6 (19.9 to 25.7)	20.4 (17.8 to 23.2)	0.3 (−3.0 to 3.8)	.81
100–199	20.2 (19.3 to 21.2)	19.0 (17.0 to 21.2)	19.7 (16.9 to 22.9)	19.4 (17.3 to 21.7)	20.7 (18.2 to 23.4)	20.8 (18.6 to 23.2)	21.8 (19.7 to 24.0)	2.7 (1.6 to 3.9)	.003
200–399	19.3 (18.6 to 20.0)	17.5 (16.1 to 19.0)	18.0 (16.0 to 20.2)	18.8 (17.1 to 20.6)	19.3 (17.5 to 21.2)	21.0 (19.0 to 23.1)	20.7 (19.2 to 22.3)	3.7 (2.4 to 5.0)	.001
≥400	18.1 (17.5 to 18.6)	17.3 (16.2 to 18.4)	16.5 (15.1 to 18.0)	18.5 (17.0 to 20.0)	18.0 (16.6 to 19.4)	18.2 (17.0 to 19.6)	19.9 (18.7 to 21.2)	2.7 (0.4 to 5.1)	.03
DLLD									
<100	18.1 (16.9 to 19.2)	17.5 (15.4 to 19.9)	17.9 (14.7 to 21.7)	16.1 (13.8 to 18.8)	20.3 (17.5 to 23.4)	18.9 (16.4 to 21.8)	17.7 (15.1 to 20.6)	1.3 (−3.8 to 6.6)	.53
100–199	16.0 (15.1 to 16.9)	15.0 (13.1 to 17.1)	16.1 (13.4 to 19.2)	16.4 (14.1 to 19.0)	14.4 (12.5 to 16.6)	16.8 (14.6 to 19.2)	17.5 (15.4 to 19.7)	2.6 (−1.4 to 6.7)	.15
200–399	14.3 (13.7 to 14.9)	13.2 (11.9 to 14.6)	14.0 (12.4 to 15.8)	14.2 (12.6 to 16.1)	14.2 (12.7 to 15.8)	15.2 (13.8 to 16.9)	14.9 (13.5 to 16.4)	2.5 (1.0 to 3.9)	.009
≥400	12.4 (12.0 to 12.9)	11.7 (10.8 to 12.7)	12.5 (11.2 to 14.0)	12.7 (11.6 to 14.0)	12.0 (10.8 to 13.2)	12.1 (11.1 to 13.2)	13.5 (12.5 to 14.7)	1.7 (−1.2 to 4.8)	.19
**Highest education level in household**									
MBDD									
Less than high school	22.4 (20.3 to 24.6)	23.3 (19.1 to 28.0)	25.9 (19.2 to 33.6)	21.1 (16.1 to 26.7)	23.2 (18.3 to 28.7)	22.9 (18.1 to 28.2)	18.5 (14.3 to 23.2)	−3.4 (−8.8 to 2.4)	.18
High school graduate	28.3 (27.2 to 29.5)	27.6 (25.2 to 30.2)	28.1 (24.6 to 31.9)	30.1 (27.3 to 33.1)	28.2 (25.4 to 31.3)	27.3 (25.0 to 29.7)	28.7 (26.3 to 31.2)	0.1 (−2.3 to 2.6)	.90
More than high school	26.3 (25.9 to 26.8)	25.0 (24.1 to 26.0)	24.7 (23.4 to 26.0)	26.2 (25.1 to 27.2)	25.9 (24.8 to 27.1)	27.5 (26.5 to 28.6)	28.7 (27.7 to 29.7)	2.9 (1.5 to 4.3)	.004
MEB									
Less than high school	17.8 (15.9 to 19.8)	17.8 (14.3 to 21.9)	22.0 (15.5 to 29.6)	14.8 (10.8 to 19.7)	19.6 (14.9 to 24.9)	17.6 (13.4 to 22.5)	15.5 (11.5 to 20.1)	−2.3 (−10.3 to 6.5)	.50
High school graduate	20.6 (19.6 to 21.6)	20.1 (18.0 to 22.3)	18.8 (16.0 to 21.8)	22.3 (19.9 to 24.8)	21.4 (18.8 to 24.2)	21.2 (19.1 to 23.4)	19.8 (17.9 to 21.9)	0.1 (−3.8 to 4.3)	.93
More than high school	19.4 (19.0 to 19.8)	18.3 (17.5 to 19.1)	17.6 (16.6 to 18.7)	19.0 (18.1 to 20.0)	19.3 (18.3 to 20.4)	20.5 (19.6 to 21.5)	21.6 (20.7 to 22.5)	3.7 (1.7 to 5.6)	.006
DLLD									
Less than high school	12.7 (11.1 to 14.5)	14.6 (10.9 to 18.9)	15.2 (9.8 to 22.0)	10.6 (7.3 to 14.8)	12.8 (9.3 to 16.9)	15.3 (11.4 to 20.0)	8.3 (5.9 to 11.3)	−6.3 (−19.2 to 8.7)	.29
High school graduate	16.9 (15.9 to 17.9)	15.6 (13.7 to 17.7)	17.2 (14.2 to 20.4)	17.1 (14.8 to 19.6)	17.8 (15.2 to 20.5)	15.5 (13.7 to 17.4)	18.2 (16.0 to 20.5)	1.5 (−3.1 to 6.4)	.42
More than high school	14.6 (14.2 to 14.9)	13.6 (12.9 to 14.3)	14.1 (13.1 to 15.2)	14.5 (13.7 to 15.4)	14.2 (13.3 to 15.1)	15.2 (14.4 to 16.0)	15.8 (15.0 to 16.6)	2.9 (1.6 to 4.1)	.003
**Primary household language**									
MBDD									
English	28.1 (27.7 to 28.6)	26.8 (25.9 to 27.8)	27.3 (25.9 to 28.7)	28.2 (27.1 to 29.3)	27.9 (26.8 to 29.1)	29.0 (28.0 to 30.0)	29.6 (28.6 to 30.6)	2.0 (1.3 to 2.6)	.001
Any other language	16.0 (14.6 to 17.5)	16.4 (13.3 to 19.8)	14.9 (10.9 to 19.6)	16.5 (13.1 to 20.4)	15.1 (11.8 to 19.0)	15.6 (12.6 to 19.0)	17.6 (14.5 to 21.1)	1.1 (−2.9 to 5.3)	.49
MEB									
English	21.0 (20.6 to 21.4)	19.8 (19.0 to 20.7)	19.7 (18.5 to 20.9)	20.8 (19.8 to 21.8)	21.2 (20.1 to 22.3)	22.1 (21.1 to 23.0)	22.2 (21.4 to 23.1)	2.5 (1.7 to 3.3)	.001
Any other language	10.9 (9.7 to 12.2)	11.5 (9.0 to 14.4)	9.5 (6.2 to 13.7)	10.7 (7.8 to 14.1)	11.0 (8.0 to 14.6)	10.8 (8.2 to 13.9)	11.8 (9.2 to 14.8)	0.8 (−3.4 to 5.1)	.65
DLLD									
English	15.8 (15.4 to 16.2)	14.7 (14.0 to 15.4)	15.7 (14.6 to 16.9)	15.7 (14.8 to 16.6)	15.6 (14.7 to 16.5)	16.4 (15.5 to 17.2)	16.7 (15.9 to 17.5)	2.4 (1.3 to 3.5)	.004
Any other language	9.4 (8.3 to 10.6)	10.2 (7.6 to 13.3)	9.5 (6.3 to 13.6)	8.6 (6.4 to 11.3)	9.4 (6.8 to 12.5)	9.3 (7.0 to 12.1)	9.5 (7.2 to 12.2)	−0.9 (−4.6 to 3.0)	.55
**Geographic classification** g									
MBDD									
Urban or suburban	26.0 (25.5 to 26.5)	24.9 (23.9 to 25.9)	25.1 (23.7 to 26.6)	26.0 (24.9 to 27.2)	25.7 (24.5 to 27.0)	27.0 (25.9 to 28.1)	27.3 (26.3 to 28.4)	1.9 (1.1 to 2.7)	.002
Rural	28.8 (27.9 to 29.7)	28.4 (26.5 to 30.3)	29.0 (26.4 to 31.8)	28.9 (26.5 to 31.4)	29.0 (26.6 to 31.5)	27.0 (24.8 to 29.3)	30.5 (28.3 to 32.8)	0.6 (−2.1 to 3.4)	.56
MEB									
Urban or suburban	19.2 (18.8 to 19.6)	18.3 (17.4 to 19.2)	17.9 (16.7 to 19.1)	18.9 (17.9 to 19.9)	19.5 (18.4 to 20.6)	20.2 (19.3 to 21.2)	20.3 (19.4 to 21.2)	2.4 (1.4 to 3.5)	.003
Rural	21.7 (20.9 to 22.6)	21.0 (19.3 to 22.7)	21.8 (19.4 to 24.2)	21.5 (19.5 to 23.7)	21.8 (19.7 to 24.1)	21.4 (19.3 to 23.6)	22.9 (20.9 to 25.0)	1.3 (−0.04 to 2.6)	.06
DLLD									
Urban or suburban	14.7 (14.4 to 15.1)	13.9 (13.1 to 14.8)	14.6 (13.4 to 15.9)	14.5 (13.6 to 15.5)	14.6 (13.6 to 15.6)	15.5 (14.6 to 16.4)	15.3 (14.5 to 16.2)	2.0 (0.8 to 3.3)	.009
Rural	15.6 (14.8 to 16.3)	15.1 (13.6 to 16.7)	16.5 (14.4 to 18.8)	15.3 (13.4 to 17.3)	15.7 (13.9 to 17.7)	13.7 (12.2 to 15.2)	17.2 (15.4 to 19.2)	0.2 (−5.5 to 6.3)	.92

Abbreviations: DLLD, developmental, learning, and language disorders; MBDD, mental, behavioral, and developmental disorder; MEB, mental, emotional, and behavioral condition.

a Calculated by linear trend test. Significant at *P* < .05.

b MBDD categories are not mutually exclusive. Children with multiple conditions are included in each applicable category.

c MEB diagnoses include attention-deficit/hyperactivity disorder, behavioral or conduct problems, anxiety problems, depression, and Tourette syndrome.

d DLLD diagnoses include autism spectrum disorder, learning disability, intellectual disability, developmental delay, and speech/other language disorder.

e Because of small subgroup sample size, annual estimates have been suppressed.

f Multiply imputed data were incorporated into the analysis for respondents with missing data for federal poverty level (18.3% of the analytic sample).

g The US. Census Bureau reviewed this data product for unauthorized disclosure of confidential information and approved the disclosure avoidance practices applied to this release: CBDRB-FY23-POP001–0105.

Among MBDD subgroups, MEBs increased overall (AAPC = 2.3; 95% CI, 1.5–3.2). Specifically, prevalence of anxiety problems (AAPC = 6.0; 95% CI, 2.8–9.2) and depression (AAPC = 5.3; 95% CI, 1.9–8.9) increased (Table 1). MEBs overall increased among females (AAPC = 4.8; 95% CI, 1.3–8.4), adolescents aged 12 to 17 years (AAPC = 3.6; 95% CI, 1.6–5.6), and non-Hispanic White children (AAPC = 2.9; 95% CI, 1.1–4.7) (Table 2). MEBs also increased among children living in urban/suburban areas (AAPC = 2.4; 95% CI, 1.4–3.5).

DLLDs also increased overall (AAPC = 1.8; 95% CI, 1.0–2.6) (Table 1). Specifically, prevalence of learning disability (AAPC = 2.9; 95% CI, 0.1–5.9), developmental delay (AAPC = 2.5; 95% CI, 0.4–4.7), and speech or other language problems (AAPC = 3.0; 95% CI, 1.5–4.7) increased (Table 1). DLLDs increased among some demographic subgroups including males (AAPC = 2.0; 95% CI, 0.5–3.5). Increases in DLLDs among other demographic subgroups followed similar patterns to those found for MBDDs overall (Table 2).

### Health care and receipt of services

Overall, from 2016 through 2021, 23.1% of US children received no preventive health care in the previous year (Table 3). Across all years, more children without MBDDs (24.9%) than with MBDDs (18.2%) received no preventive medical care in the previous year. Unmet health care needs in the past 12 months among children overall increased by an average of 5% annually during the study period. Specifically, unmet health care needs increased by 5.9% (95% CI, 1.7–10.2) annually among children with MEBs. Unmet health care needs among children with DLLDs were consistently higher than unmet health care needs among children with no MBDDs and remained stable over the study period (Table 3). The prevalence of parent-reported adequacy of health insurance did not change significantly from 2016 through 2021. Receipt of mental and/or developmental services for each calendar year among children with MBDDs also remained stable over the study period: each year during the study period, approximately 60% of children with MBDDs (MEB, 62.0%; DLLD, 69.1%) received mental and/or developmental services in the prior 12 months. Receipt of special education or intervention services remained stable over the time period for children with MBDDs, with 33.3% of children with MBDDs (MEB, 30.2%; DLLD, 51.7%) receiving these services (Table 3).

**Table 3:** Prevalence of Health Care, Family, and Community Indicators Among Children Aged 3–17 Years, Overall and by MBDD Status, National Survey of Children’s Health, 2016–2021

Variable		2016–2021, % (95% CI)	2016, % (95% CI)	2017, % (95% CI)	2018, % (95% CI)	2019, % (95% CI)	2020, % (95% CI)	2021, % (95% CI)	Average Annual % Change, (95% CI)	*P* value^a^
**Health care and receipt of services**										
Lacked preventive medical care, last 12 months^c^	Total	23.1 (22.6 to 23.6)	19.3 (18.4 to 20.3)	19.6 (18.2 to 21.0)	—b	17.9 (16.8 to 19.0)	23.8 (22.8 to 24.9)	27.1 (26.0 to 28.1)	7.2 (−1.1 to 16.2)	.07
	MBDD	18.2 (17.3 to 19.0)	15.3 (13.7 to 17.0)	15.8 (13.2 to 18.6)	—b	13.6 (12.0 to 15.4)	17.0 (15.4 to 18.8)	20.9 (19.1 to 22.9)	5.8 (−5.0 to 17.8)	.20
	MEB	17.6 (16.7 to 18.5)	15.1 (13.3 to 17.2)	15.4 (12.5 to 18.7)	—b	13.5 (11.6 to 15.5)	16.4 (14.5 to 18.4)	19.4 (17.5 to 21.5)	4.8 (−4.4 to 14.8)	.21
	DLLD	17.4 (16.3 to 18.5)	15.3 (13.0 to 17.7)	14.3 (11.1 to 18.1)	—b	12.0 (10.1 to 14.2)	17.2 (15.0 to 19.6)	20.6 (18.2 to 23.1)	6.5 (−7.9 to 23.2)	.26
	No MBDD	24.9 (24.3 to 25.5)	20.7 (19.6 to 21.9)	20.8 (19.3 to 22.5)	—b	19.5 (18.1 to 20.9)	26.4 (25.1 to 27.7)	29.4 (28.1 to 30.7)	7.4 (−1.2 to 16.8)	.07
Unmet health care needs, last 12 months^d^	Total	3.7 (3.5 to 4.0)	3.3 (2.9 to 3.8)	3.6 (2.9 to 4.4)	3.5 (3.0 to 4.0)	3.4 (2.8 to 3.9)	4.5 (3.9 to 5.2)	4.1 (3.7 to 4.6)	5.0 (0.3 to 1.0)	.04
	MBDD	8.1 (7.5 to 8.7)	7.4 (6.4 to 8.5)	7.1 (5.4 to 9.0)	7.4 (6.2 to 8.7)	8.5 (6.9 to 10.4)	9.1 (7.8 to 10.7)	8.9 (7.8 to 10.1)	4.7 (1.6 to 7.9)	.01
	MEB	9.3 (8.7 to 10.0)	8.3 (7.1 to 9.6)	7.9 (6.2 to 9.8)	8.3 (6.9 to 9.8)	10.2 (8.1 to 12.6)	10.7 (9.0 to 12.6)	10.4 (9.0 to 11.9)	5.9 (1.7 to 10.2)	.02
	DLLD	8.4 (7.6 to 9.2)	7.8 (6.3 to 9.5)	8.2 (5.7 to 11.3)	8.8 (7.0 to 10.9)	8.5 (6.3 to 11.1)	9.1 (7.3 to 11.0)	7.9 (6.7 to 9.2)	0.3 (−4.0 to 4.9)	.84
	No MBDD	2.2 (1.9 to 2.4)	1.9 (1.5 to 2.4)	2.4 (1.7 to 3.3)	2.1 (1.6 to 2.6)	1.5 (1.2 to 1.9)	2.8 (2.2 to 3.5)	2.3 (1.8 to 2.8)	4.3 (−7.9 to 18.1)	.40
Health insurance adequacy^e^	Total	26.7 (26.2 to 27.1)	25.5 (24.6 to 26.4)	27.0 (25.7 to 28.3)	27.0 (25.9 to 28.1)	28.5 (27.3 to 29.8)	26.7 (25.7 to 27.8)	25.3 (24.4 to 26.3)	0.0 (−3.1 to 3.2)	.997
	MBDD	31.8 (30.9 to 32.7)	29.2 (27.5 to 31.1)	32.3 (29.6 to 35.1)	32.2 (30.0 to 34.4)	34.4 (32.1 to 36.7)	31.7 (29.8 to 33.6)	30.9 (29.1 to 32.7)	0.9 (−2.7 to 4.7)	.54
	MEB	33.5 (32.5 to 34.5)	30.4 (28.3 to 32.5)	33.0 (30.0 to 36.2)	34.4 (31.9 to 37.1)	36.4 (33.6 to 39.2)	33.6 (31.4 to 35.9)	33.0 (30.9 to 35.1)	1.6 (−2.1 to 5.4)	.31
	DLLD	31.1 (29.9 to 32.3)	27.9 (25.6 to 30.4)	33.1 (29.3 to 37.1)	32.7 (29.7 to 35.7)	33.6 (30.6 to 36.8)	29.9 (27.5 to 32.4)	29.3 (26.9 to 31.7)	0.1 (−5.2 to 5.7)	.95
	No MBDD	24.8 (24.3 to 25.3)	24.2 (23.1 to 25.2)	25.1 (23.6 to 26.7)	25.1 (23.8 to 26.4)	26.4 (25.0 to 27.9)	24.9 (23.7 to 26.1)	23.1 (22.0 to 24.3)	−0.3 (−3.3 to 2.7)	.79
Received mental and/or developmental services, last 12 months^f^	Total	18.9 (18.5 to 19.2)	18.0 (17.3 to 18.8)	17.7 (16.6 to 18.9)	18.0 (17.1 to 18.9)	19.2 (18.2 to 20.2)	20.0 (19.1 to 20.9)	20.3 (19.4 to 21.1)	2.8 (1.4 to 4.2)	.005
	MBDD	60.0 (59.1 to 60.9)	58.8 (56.7 to 60.8)	57.3 (54.3 to 60.2)	59.0 (56.6 to 61.3)	62.3 (60.0 to 64.5)	61.5 (59.5 to 63.5)	60.9 (58.9 to 62.9)	1.1 (−0.3 to 2.5)	.10
	MEB	62.0 (60.9 to 63.1)	61.3 (59.0 to 63.6)	58.3 (54.9 to 61.7)	59.9 (57.2 to 62.6)	65.3 (62.7 to 67.9)	63.8 (61.5 to 66.1)	62.8 (60.6 to 65.1)	1.1 (−1.1 to 3.3)	.25
	DLLD	69.1 (67.9 to 70.3)	67.6 (64.8 to 70.3)	68.9 (65.1 to 72.5)	68.7 (65.8 to 71.6)	70.2 (67.1 to 73.1)	71.1 (68.6 to 73.5)	68.2 (65.5 to 70.8)	0.5 (−0.9 to 1.8)	.39
	No MBDD	4.1 (3.9 to 4.4)	4.2 (3.7 to 4.7)	4.2 (3.6 to 4.8)	3.2 (2.8 to 3.7)	3.9 (3.5 to 4.4)	4.6 (4.1 to 5.2)	4.7 (4.1 to 5.3)	3.4 (−6.4 to 14.3)	.40
Received special education or intervention services, last 12 months^g^	Total	9.8 (9.5 to 10.1)	8.9 (8.4 to 9.5)	9.6 (8.7 to 10.6)	9.6 (8.9 to 10.4)	9.7 (9.0 to 10.4)	10.6 (9.9 to 11.3)	10.4 (9.7 to 11.1)	3.3 (1.5 to 5.2)	.007
	MBDD	33.3 (32.4 to 34.2)	31.3 (29.5 to 33.2)	33.6 (30.7 to 36.6)	33.8 (31.5 to 36.1)	33.9 (31.6 to 36.2)	34.6 (32.6 to 36.7)	32.6 (30.8 to 34.5)	1.0 (−1.4 to 3.4)	.32
	MEB	30.2 (29.2 to 31.2)	28.3 (26.2 to 30.3)	30.2 (27.0 to 33.4)	28.9 (26.7 to 31.2)	32.1 (29.4 to 34.8)	32.7 (30.4 to 35.2)	28.9 (26.9 to 30.9)	1.3 (−2.7 to 5.5)	.42
	DLLD	51.7 (50.4 to 53.0)	48.9 (46.1 to 51.8)	51.9 (47.8 to 56.0)	53.3 (50.1 to 56.4)	52.0 (48.7 to 55.3)	52.7 (49.9 to 55.6)	51.2 (48.4 to 53.9)	0.8 (−1.2 to 2.8)	.33
	No MBDD	1.4 (1.3 to 1.5)	1.4 (1.1 to 1.7)	1.3 (1.0 to 1.8)	1.0 (0.7 to 1.3)	1.2 (0.9 to 1.4)	1.7 (1.4 to 2.1)	1.8 (1.4 to 2.4)	8.6 (−7.3 to 27.3)	.22
**Parent and family well-being**										
≥1 parent with fair/poor mental health^h^	Total	8.1 (7.8 to 8.4)	7.6 (7.0 to 8.2)	7.6 (6.8 to 8.6)	7.1 (6.4 to 7.8)	7.9 (7.1 to 8.7)	8.8 (8.1 to 9.5)	9.6 (8.9 to 10.3)	5.4 (1.0 to 10.0)	.03
	MBDD	14.7 (14.0 to 15.5)	13.4 (12.0 to 15.0)	14.9 (12.7 to 17.3)	12.9 (11.2 to 14.7)	14.1 (12.2 to 16.2)	15.9 (14.2 to 17.6)	16.8 (15.3 to 18.5)	4.5 (0.6 to 8.6)	.03
	MEB	16.3 (15.4 to 17.1)	15.6 (13.8 to 17.5)	16.8 (14.2 to 19.5)	14.0 (12.3 to 15.8)	15.5 (13.1 to 18.0)	17.5 (15.5 to 19.7)	18.0 (16.3 to 19.8)	3.1 (−1.9 to 8.4)	.16
	DLLD	14.3 (13.3 to 15.3)	12.0 (10.3 to 13.9)	14.2 (11.2 to 17.7)	13.4 (10.9 to 16.2)	13.7 (11.5 to 16.2)	15.0 (13.0 to 17.2)	17.0 (15.0 to 19.2)	6.4 (3.1 to 9.9)	.006
	No MBDD	5.7 (5.4 to 6.0)	5.6 (5.0 to 6.2)	5.1 (4.3 to 6.1)	5.0 (4.4 to 5.6)	5.6 (4.9 to 6.4)	6.2 (5.5 to 6.9)	6.7 (6.1 to 7.5)	4.7 (−0.4 to 10.0)	.06
Household economic stress, lifetime^i^	Total	14.2 (13.7 to 14.7)	—b	—b	15.7 (14.7 to 16.7)	15.8 (14.8 to 16.9)	13.1 (12.3 to 14.0)	12.2 (11.4 to 13.1)	−9.0 (−18.5 to 1.6)	.07
	MBDD	21.6 (20.6 to 22.6)	—b	—b	23.7 (21.6 to 25.9)	24.5 (22.3 to 26.9)	20.9 (19.0 to 22.9)	17.6 (16.0 to 19.2)	−10.0 (−22.1 to 4.1)	.09
	MEB	22.9 (21.8 to 24.1)	—b	—b	24.6 (22.4 to 26.8)	26.5 (23.8 to 29.3)	22.3 (20.0 to 24.6)	18.7 (16.9 to 20.6)	−9.1 (−23.7 to 8.3)	.14
	DLLD	23.6 (22.2 to 25.0)	—b	—b	26.1 (23.1 to 29.4)	27.5 (24.3 to 30.9)	22.3 (19.8 to 25.0)	18.7 (16.5 to 21.0)	−11.5 (−25.7 to 5.6)	.10
	No MBDD	11.5 (11.0 to 12.0)	—b	—b	12.8 (11.6 to 14.0)	12.8 (11.6 to 14.0)	10.2 (9.4 to 11.2)	10.2 (9.2 to 11.1)	−8.7 (−20.5 to 4.9)	.11
**Community support and safety**										
Lacking neighborhood support^j^	Total	44.0 (43.5 to 44.5)	45.5 (44.4 to 46.7)	42.6 (41.0 to 44.2)	43.5 (42.2 to 44.8)	45.5 (44.2 to 46.9)	43.9 (42.7 to 45.1)	42.9 (41.7 to 44.0)	−0.7 (−2.4 to 1.1)	.34
	MBDD	49.7 (48.8 to 50.7)	52.4 (50.3 to 54.4)	48.5 (45.5 to 51.5)	50.2 (47.8 to 52.6)	50.1 (47.7 to 52.5)	49.5 (47.4 to 51.6)	48.0 (45.9 to 50.0)	−1.3 (−2.7 to 0.0)	.05
	MEB	50.6 (49.5 to 51.6)	52.7 (50.4 to 55.1)	49.6 (46.2 to 53.0)	51.6 (48.9 to 54.3)	50.1 (47.3 to 52.9)	50.8 (48.4 to 53.2)	48.7 (46.3 to 51.0)	−1.2 (−2.5 to 0.1)	.07
	DLLD	51.1 (49.8 to 52.4)	52.9 (50.1 to 55.8)	50.2 (46.1 to 54.3)	49.8 (46.5 to 53.0)	53.5 (50.3 to 56.7)	51.1 (48.4 to 53.9)	49.2 (46.4 to 52.0)	−0.9 (−3.0 to 1.2)	.31
	No MBDD	41.9 (41.3 to 42.6)	43.2 (41.9 to 44.5)	40.6 (38.7 to 42.5)	41.1 (39.5 to 42.7)	43.9 (42.3 to 45.6)	41.8 (40.4 to 43.2)	4.9 (39.5 to 42.2)	−0.5 (−2.6 to 1.5)	.51
Lacking perceived neighborhood safety^k^	Total	5.3 (5.0 to 5.6)	6.1 (5.4 to 6.8)	4.9 (4.2 to 5.7)	4.7 (4.1 to 5.5)	5.4 (4.7 to 6.1)	5.6 (5.0 to 6.3)	5.0 (4.4 to 5.6)	−2.2 (−8.2 to 4.3)	.39
	MBDD	7.1 (6.6 to 7.8)	7.7 (6.3 to 9.3)	7.3 (5.6 to 9.3)	5.7 (4.6 to 7.0)	8.5 (6.9 to 10.4)	7.8 (6.4 to 9.4)	5.8 (4.7 to 7.1)	−2.4 (−12.9 to 9.3)	.58
	MEB	7.5 (6.9 to 8.3)	8.1 (6.6 to 10.0)	7.2 (5.6 to 9.0)	6.8 (5.4 to 8.5)	9.1 (7.1 to 11.5)	8.1 (6.5 to 10.0)	6.0 (4.8 to 7.4)	−2.4 (−11.4 to 7.5)	.53
	DLLD	7.8 (7.0 to 8.7)	7.5 (5.8 to 9.7)	8.0 (5.5 to 11.2)	5.8 (4.5 to 7.4)	10.2 (7.9 to 13.0)	8.8 (6.8 to 11.1)	6.7 (5.1 to 8.6)	1.5 (−13.5 to 19.1)	.81
	No MBDD	4.6 (4.3 to 4.9)	5.5 (4.8 to 6.4)	4.1 (3.3 to 4.9)	4.4 (3.6 to 5.3)	4.2 (3.5 to 5.1)	4.8 (4.1 to 5.6)	4.7 (4.0 to 5.5)	−1.9 (−8.9 to 5.7)	.52

Abbreviations: DLLD, developmental, learning, and language disorders; MBDD, mental, behavioral, and developmental disorder; MEB, mental, emotional, and behavioral conditions.

a Calculated by linear trend test. Significant at *P* < .05.

b Data suppressed because wording of this question for these years differed substantially.

c The child did not see a health care professional for sick-child care, well-child check-ups, physical exams, hospitalizations, or any other kind of medical care, during the past 12 months.

d The child did not receive needed health care, during the past 12 months.

e The child had no health insurance or the child’s health insurance coverage was inadequate to meet the child’s needs, during the past 12 months.

f The child received mental and/or developmental services, during the past 12 months.

g The child received services through a special education or early intervention plan. This group is a subset of those children who received any mental and/or developmental services during the last 12 months.

h Parent (both parents if 2 primary caregivers) reported fair/poor (vs good/very good/excellent) mental health.

i Parent reported that it was that it was difficult to get by on the family’s income somewhat/very often (vs. never/rarely).

j Parent reported response of “definitely disagree/somewhat disagree” (vs. somewhat/definitely agree) when asked whether people in the neighborhood help each other out, watch out for each other’s children, and know where to go for help in their community when they encounter difficulties.

k Parent reported response of “definitely disagree/somewhat disagree” (vs somewhat/definitely agree) when asked whether the child is safe in their neighborhood.

### Parent and family well-being

Poor parental mental health increased overall (AAPC = 5.4; 95% CI, 1.0–10.0), with 9.6% of parents reporting poor mental health in 2021. The percentage of parents of children with DLLDs reporting poor mental health had an average annual increase of 6.4% during the study period (95% CI, 3.1–9.9). The percentage of parents of children with MEBs reporting poor mental health remained stable. The AAPCs for household economic stress were similar across groups from 2018 through 2021, ranging from −11.5 to −8.7. Although AAPCs for household economic stress decreased over the study period, the decreases were not significant. Each year, a higher percentage of parents of children with MBDDs compared with those without MBDDs reported poor mental health (eg, 2021: 16.8% MBDD, 6.7% no MBDD) and economic stress (eg, 2021: 17.6% MBDD, 10.2% no MBDD) (Table 3).

### Community support and safety

Among all children, 44.0% had parents who reported lacking social support in their neighborhood, and 5.3% had parents who perceived their neighborhood to be unsafe. These indicators did not change significantly between 2016 and 2021. Lack of neighborhood support (MBDD: 49.7%; no MBDD: 41.9%) and lack of perceived neighborhood safety (MBDD: 7.1%; no MBDD: 4.6%) were higher among children with MBDDs than without during the study period (Table 3).

## Discussion

The goal of our study was to examine trends in lifetime prevalence of diagnosed mental, behavioral, and developmental disorders and in the health care, family, and community factors associated with these disorders from 2016 through 2021. Although some of our findings about MBDD prevalence are consistent with other reports showing rising prevalence among some MBDDs before (4,12) and during the pandemic (12–14), our study extends these findings by examining lifetime prevalence, a more comprehensive list of disorders, and contextual factors associated with disparities in MBDD prevalence.

Between 2016 and 2021, lifetime prevalence of numerous parent-reported childhood MBDDs increased overall and among some demographic subgroups. Specifically, the prevalence of anxiety problems, depression, learning disability, developmental delay, and speech or other language problems increased during this time period, which included years before and during the COVID-19 pandemic. The lifetime prevalence of MEBs increased with average annual percentage changes of approximately 5% among females, approximately 4% among adolescents, and approximately 3% to 4% among children living in poverty, highlighting specific groups who may benefit from focused intervention and treatment efforts to offset ongoing effects of chronic mental, behavioral, and developmental problems. Pediatric care providers are often the first point of contact for children experiencing mental health concerns, but many providers report that they lack the resources and training to identify or manage children’s mental health problems (15). Improving mental health education and training for health care professionals, providing mental health practice tools and resources, and developing innovative collaborations between clinicians working in behavioral health and pediatric care providers can help address the mental health needs of children (16,17).

MBDDs may be associated with the contexts in which children grow and develop and the factors that determine access to health care and community resources (2). Monitoring changes in the prevalence of these contexts and factors can help identify areas where intervention and prevention efforts are needed. During the 2016 to 2021 period, some indicators known to be associated with MBDDs increased, while others remained stable but were higher among children with MBDDs than those without. The prevalence of poor parental mental health increased overall, especially among parents of children with DLLDs. In 2021, approximately 2.5 times as many children with DLLDs as those without MBDDs had a parent with poor mental health. Parents and caregivers with poor mental health may have greater difficulty responding to their child’s needs and coping with the demands of parenting (18). The COVID-19 pandemic exacerbated parenting challenges, particularly for parents of children with disabilities (7,8). Improving parents’ mental health, particularly parents of children with MBDDs, can help parents support their own health and their children’s mental health and well-being (2,5). Various federal programs and resources to reduce stress and promote parental mental health are available, such as support for early childhood (19,20), enhancing well-being in children (21), and supporting adult well-being (22). Health care providers can support families by reinforcing strengths and identifying risks (17).

Families living in rural areas disproportionately experience gaps in access to health and mental health care, partly because of a shortage of mental health clinicians practicing in rural areas (23). We found that although the prevalence of MBDDs among children living in urban areas increased, the prevalence of parent-reported MBDDs among children in rural areas was higher overall than among children in urban or suburban areas. This finding is consistent with previous reports focusing on younger children that showed higher MBDD prevalence in rural areas (24). Increasing availability and access to evidence-based mental and behavioral health care through telemedicine, integration of behavioral health and primary care, and school-based care may improve outcomes for children living in rural areas (16,25,26).

Preventive care visits are opportunities for health promotion, identification of children at risk for MBDDs and those in need of services, and early intervention (4,7). Other studies have demonstrated that a substantial decrease in preventive care visits for children in the US occurred during the COVID-19 pandemic (7,27). Our finding of a 5.9% average annual increase, beginning before the pandemic and continuing through 2021, in the percentage of parents of children with MEBs who reported unmet health care needs may be associated with the decreases in preventive care resulting from the pandemic reported in other studies (7). Strengthening policies to promote the affordability, accessibility, and use of health care services may increase the use of preventive care, particularly among children living in rural areas (8), for whom prevalence of MBDDs was higher. In addition, efforts to support families and developmental health through parent-engaged developmental monitoring, for example, the “*Learn the Signs. Act Early*” program (28), may be an opportunity to identify concerns earlier and support parents in talking with clinicians.

Although receipt of mental and/or developmental services remained stable among children with MBDDs and although many children with MBDDs received services during 2016–2021, it is notable that each year during the study period approximately 40% did not receive these services in the prior 12 months, pointing to possible systemic gaps in intervention that can be addressed to ensure equitable access to appropriate services in a wide range of community contexts (7,12,29). Timely receipt of these services can improve mental and developmental outcomes (2,4,5).

More parents of children with than without MBDDs reported household economic stress during the child’s lifetime, lacking neighborhood support, and perceived lack of neighborhood safety. Economic stability, social support, and neighborhood safety are social determinants of health that can promote the overall well-being of children, families, and communities (4,5,8,12). Policies that support income security for families (eg, Earned Income Tax Credits, Supplemental Security Income, and Medicaid Home and Community-Based Services waivers for children with disabilities, paid family leave) can improve child health and well-being on a population level (5,7,30).

### Limitations

Our study had limitations. First, the presence of MBDDs was based on parent report of a diagnosis and may be limited by parent recall and willingness to report; their presence was not verified by clinical assessment or review of medical records and is affected by access to diagnosis. Second, the data are cross-sectional, and direction of causality between MBDDs and associated indicators cannot be determined. Third, findings may underestimate increased MBDD prevalence among those sociodemographic subgroups with limited access to health care. Furthermore, nonresponse bias is possible, but US Census Bureau analyses did not show evidence of systematic bias after sampling weights were applied (31). Fourth, linear test for trends is sensitive to sample size; demographic groups with small sample sizes may lack the power to detect differences of similar magnitude to demographic groups with large sample sizes. Future analyses with larger samples of demographic subgroups and data collection of parent-reported symptoms and diagnoses may provide important information about health equity and potential differences associated with factors such as race or ethnicity. Fifth, the analytic approach we used to calculate average annual percentage changes assumed linear trends; other approaches to trend analysis with more years of data could investigate nonlinear trends, including changes concurrent with the COVID-19 pandemic and whether those changes continue or were transient.

### Conclusion

The increasing prevalence of certain MBDDs (specifically anxiety, depression, learning disability, developmental delay, and speech or language disorder) and MBDD-associated indicators during 2016-2021 (which included years before and during the COVID-19 pandemic) suggest opportunities to promote well-being and improve children’s mental health. Improved pediatric training for health care providers may help clinicians identify and support patients with MBDDs. With sufficient training, health care providers can play a significant role in addressing concerns about MBDDs in childhood and supporting healthy development through preventive care, supportive interventions, and referrals (17,32). Developing tailored and comprehensive public health prevention and intervention efforts may include focused attention on the mental health of adolescents and girls, early intervention for children with DLLDs (28), and support for families of children with MBDDs, particularly those living in poverty (30). Enacting policies that address contextual factors, such as economic stability and equitable access to health care services, including policies that support location-based pediatric mental health services (eg, school-based services, integrated care models) and affordability (eg, public insurance, mental health parity) can advance and reinforce prevention efforts (5). Upstream prevention efforts by schools, communities, and health care providers, such as providing safe and supportive environments (33,34), fostering social connections (35), and supporting parents and children with available emotional well-being resources (21), can promote mental health and improve outcomes for all children.

## Appendix

**Appendix Table:** Wording of National Survey of Children’s Health, by Survey Administration Year

Variable	Administration year(s)	Survey question
**Mental, behavioral, and developmental disorder**		
Attention deficit/hyperactivity disorder	2016–2021	Has a doctor or other health care provider ever told you that this child has [specified disorder]?
Anxiety problems		
Depression		
Tourette syndrome		
Autism spectrum disorder		
Behavioral and conduct problems	2016–2021	Has a doctor, other health care provider, or educator ever told you that this child has (specified disorder)?
Developmental delay		
Intellectual disability		
Learning disability		
Speech or other language disorder		
**Health care and receipt of services**		
Lacked preventive medical care, last 12 months	Composite of:	
	2016–2017, 2019–2021	During the past 12 months, did this child see a doctor, nurse, or other health care professional for sick-child care, well-child check-ups, physical exams, hospitalizations, or any other kind of medical care?
	Or	
	2018	During the past 12 months, did this child see a doctor, nurse, or other health care professional for medical care (for example, preventive care, sick care, hospitalizations)?^a^
	And	
	2016–2021	During the past 12 months, how many times did this child visit a doctor, nurse, or other health care professional to receive a preventive check-up? A preventive check-up is when this child was not sick or injured.
Unmet health care needs, last 12 months	2016–2021	During the past 12 months, was there any time when this child needed health care but it was not received? Health care includes medical care, dental care, vision care, and mental health services.
Health insurance adequacy	2016–2021	Composite of: During the past 12 months was this child ever covered by any kind of health insurance or health coverage plan? How often does this child’s health insurance offer benefits or cover services that meet this child’s needs? How often does this child’s health insurance allow them to see the health care providers they^b^ need? How often are [out-of-pocket medical or health-related] costs reasonable?
Received mental and/or developmental services, last 12 months	2016–2021	Composite of: Has this child ever had a special education or early intervention plan? Children receiving these services often have an Individualized Family Service Plan or Individualized Education Plan. Is this child currently receiving services under one of these plans? Has this child ever received special services to meet their^b^ developmental needs such as speech, occupational, or behavioral therapy? Is this child currently receiving these special services? During the past 12 months, has this child received any treatment or counseling from a mental health professional? Mental health professionals include psychiatrists, psychologists, psychiatric nurses, and clinical social workers. At any time during the past 12 months, did this child receive behavioral treatment for ADD or ADHD, such as training or an intervention that you or this child received to help with their^b^ behavior? At any time during the past 12 months, did this child receive behavioral treatment for autism, autism spectrum disorder (ASD), Asperger Disorder or Pervasive Development Disorder (PDD), such as training or an intervention that you or this child received to help with their^b^ behavior?
Received special education or intervention services, last 12 months	2016–2021	Composite of: Has this child ever had a special education or early intervention plan? Children receiving these services often have an Individualized Family Service Plan or Individualized Education Plan. Is this child currently receiving services under one of these plans?
**Parent and family well-being**		
≥1 parent with fair/poor mental health	Composite of	
	2016–2021	In general, how is your mental or emotional health?
	And	
	2016–2017	In general, how is Adult 2's mental or emotional health? Or
	Or	
	2018	In general, how is this primary caregiver's [caregiver 2] mental or emotional health?
	Or	
	2019	In general, how is Caregiver 2’s mental or emotional health?
	Or	
	2020–2021	In general, how is this caregiver’s [caregiver 2] mental or emotional health?
Household economic stress, lifetime	2016–2017	Since this child was born, how often has it been very hard to get by on your family's income - hard to cover the basics like food or housing?^c^
	2018–2021	Since this child was born, how often has it been very hard to cover the basics, like food or housing, on your family’s income?
**Community support and safety**		
Lacking neighborhood support	2016–2021	Composite of: To what extent do you agree with these statements about your neighborhood or community: People in this neighborhood help each other out. We watch out for each other's children in this neighborhood. When we encounter difficulties, we know where to go for help in our community.
Lacking perceived neighborhood safety	2016–2021	To what extent do you agree with these statements about your neighborhood or community: This child is safe in our neighborhood.

^a^ Data for lack of preventive medical care for 2018 are not included in the analysis because of different question wording for that year.

^b^ From 2016–2019, he or she/his or her pronouns were used for these questions; beginning in 2020, they/their pronouns were used.

^c^ Data for household economic stress for 2016 and 2017 are not included in analyses due to different question wording for those years.
